# Venous Thromboembolism Following Colorectal Surgery for Suspected or Confirmed Malignancy

**DOI:** 10.1155/2011/828030

**Published:** 2011-05-26

**Authors:** Brenton Sanderson, Kerry Hitos, John P. Fletcher

**Affiliations:** Department of Surgery, University of Sydney, Westmead Hospital, Westmead, NSW 2145, Australia

## Abstract

Surgery for colorectal cancer conveys a high risk of venous thromboembolism (VTE). The effect of thromboprophylactic regimens of varying duration on the incidence of VTE was assessed in 417 patients undergoing surgery between 2005 and 2009 for colorectal cancer. Low-dose unfractionated heparin (LDUH) was used in 52.7% of patients, low-molecular-weight heparin (LMWH) in 35.3%, and 10.7% received LDUH followed by LMWH. Pharmacological prophylaxis was continued after hospitalisation in 31.6%. Major bleeding occurred in 4% of patients. The 30-day mortality rate was 1.9%. The incidence of symptomatic VTE from hospital admission for surgery to 12 months after was 2.4%. There were no in-hospital VTE events. The majority of events occurred in the three-month period after discharge, but there were VTE events up to 12 months, especially in patients with more advanced cancer and multiple comorbidities.

## 1. Introduction

Major surgery conveys a significant risk of venous thromboembolism (VTE), a condition that encompasses both deep vein thrombosis (DVT) and pulmonary embolism (PE). The first-time incidence of VTE in the general population is approximately 100 persons per 100,000 per year [1]. VTE is a common cause of morbidity and mortality in surgical patients which is preventable in the majority of cases with appropriate prophylaxis [1, 2]. There are numerous risk factors for developing VTE and, importantly, for colorectal cancer surgery patients, include increasing age, history of VTE, malignancy and its treatment, and surgery duration [3–5]. The presence of these risk factors places colorectal cancer surgery patients at an increased risk of VTE compared to general surgical patients [5, 6]. A retrospective analysis examined VTE in colorectal cancer patients, 70% of whom underwent surgery, and found a VTE incidence of 3.1% (2,100 patients) at two years [6]. The incidence rate was found to decrease over the two-year period, demonstrating the importance of VTE prophylaxis in the period after diagnosis and perioperatively [6]. The strongest predictors for VTE occurrence in this patient group were found to be the presence of metastatic disease and multiple co-morbidities [6].

In the American College of Chest Physicians (ACCP) evidence-based clinical practice guidelines, colorectal cancer surgery patients are judged as having a high risk of VTE and are recommended to receive both anticoagulant and mechanical VTE prophylaxis unless contraindicated [7]. For anticoagulant prophylaxis, colorectal cancer surgery patients are recommended to receive low-molecular-weight heparin (LMWH), low-dose unfractionated heparin (LDUH) three times daily, or fondaparinux until discharge from hospital [7]. Mechanical prophylaxis is recommended in the form of graduated compression stockings (GCS) and/or the use of an intermittent pneumatic compression (IPC) device [7]. In addition, the 2008 ACCP guidelines suggest consideration of VTE prophylaxis with LMWH being continued after hospital discharge for up to 28 days for patients who have undergone major cancer surgery [7].

In 2009, the National Health and Medical Research Council (NHMRC) of Australia produced evidence-based clinical practice guidelines for the prevention of VTE to improve the application of VTE prophylaxis recommendations in Australian hospitals [8]. These guidelines recommend the appropriate use of anticoagulant prophylaxis for at least seven to 10 days postoperatively in the form of LMWH or LDUH and, if contraindicated, the use of GCS [8]. The use of extended thromboprophylaxis with LMWH for up to 28 days after colorectal cancer surgery is also recommended for consideration [8].

The current study was undertaken to review the effect of different types and duration of thromboprophylactic modalities on VTE incidence and to evaluate the incidence and risk factors for bleeding complications in patients undergoing surgery for colorectal cancer.

## 2. Materials and Methods

From January 1, 2005 to December 31, 2009, 417 patients who underwent colorectal surgery for suspected or confirmed malignancy were identified from the Westmead Hospital Department of Surgery database registry. All patient records were analysed retrospectively for the occurrence of DVT and/or PE, 30-day mortality, and hospital readmission within three months and twelve months of surgery.

From this study population, a computer-generated random sample of 150 patients, 30 per year, was selected using Statistical Product and Service Solutions version 16.0 (SPSS Inc., Chicago, IL, USA). The medical records of these patients were then reviewed for the following variables: patient demographics, diagnosis and co-morbidities, preoperative haemoglobin level, operative and anaesthetic details, pre- and postoperative VTE prophylaxis, and postoperative complications, including major bleeding.

Symptomatic DVT was confirmed by documentation of positive duplex ultrasonography for DVT and for symptomatic PE by high probability ventilation perfusion lung scan or CT pulmonary angiography. Readmission for VTE was recorded when a patient having been discharged from hospital was readmitted with VTE symptoms within a three-month period with imaging confirming a diagnosis of DVT and/or PE.

Pharmacological prophylaxis was LMWH enoxaparin (Clexane/Lovenox; Sanofi-aventis, Paris, France) administered subcutaneously (20 mg or 40 mg daily) preoperatively and/or postoperatively or LDUH subcutaneously (5,000 IU) two or three times daily preoperatively and/or postoperatively. Pharmacological prophylaxis administration was expected to continue for seven to 10 days according to ACCP guidelines [7]. GCS and/or IPC devices were applied immediately preoperatively and continued intraoperatively and postoperatively. Major bleeding complications were defined as non anaemic patients preoperatively (haemoglobin > 120 g/L for females and > 130 g/L for males) who experienced bleeding requiring transfusion of greater than two units of blood during their hospital admission, cessation of heparin, or reoperation [9]. Days to ambulation was considered as the number of days from date of operation to the date of first documentation by physiotherapist or nursing staff that the patient was ambulatory.

### 2.1. Statistical Analysis

Data was entered into a specifically designed Clinical Reporting Systems database version 1.1 ma (G.E. Medical System Pty Ltd., Australia). Continuous data is given as the median and interquartile range (IQR, range from the 25th to the 75th percentile). Continuous data groups were compared using the two-tailed Mann Whitney *U* test. Statistical significant differences were considered at the *P* < .05 level and where possible 95% confidence intervals (CI) are presented.

## 3. Results

### 3.1. Patient Characteristics

The median patient age was 67 (IQR: 58.3–76) with a male-to-female ratio of 1 : 1. The median length of stay (LOS) was 10 days (IQR: 7–14), decreasing from 13 days (IQR: 10.3–19.8) in 2005 to seven days (IQR: 6–11) in 2009. The LOS for laparoscopic and open procedure patients was seven days (IQR: 6–10) and 11 days (IQR: 9–15), respectively (*P* < .0001). In terms of VTE risk factors, 98% (95% CI: 94.3–99.3%) of patients had confirmed malignancy with malignant neoplasm of the rectum being the most common site, accounting for 27.3% (95% CI: 20.8–34.9%) of all patients. 72.7% (95% CI: 65.0–79.2%) of patients had a cancer stage of three or higher. The median number of co-morbidities was three (IQR: 2–4.8), with 5.3% (95% CI: 2.7–10.2%) of patients having a history of VTE. 44% (95% CI: 36–52%) had an American Society of Anaesthesiology (ASA) score of three (indicating severe systemic disease) and 47% (95% CI: 39.9–55.6%) a score of two (mild systemic disease).

### 3.2. Operative and Anaesthetic Details

Anterior resection was the most common procedure performed, accounting for 45.3% (95% CI: 37.6–53.3%) of all procedures, followed by right hemicolectomy in 28.7% (95% CI:22.0–36.4%), total colectomy in 8.7% (95% CI: 5.3–14.1%), and abdominoperineal resection in 7.3% (95% CI: 4.1–12.7%). A further 10% (95% CI: 6.2–15.8%) included other colorectal surgery such as Hartmann's procedure and left hemicolectomy. Overall, 26% (95% CI: 19.6–33.6%) of operations were performed laparoscopically. 

**Figure 1:**
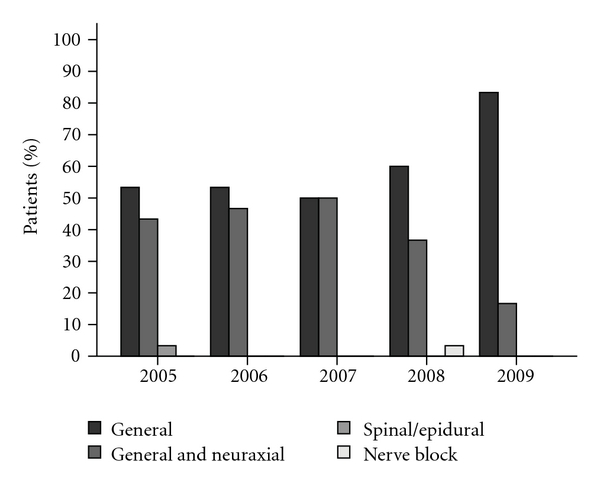
Anaesthesia usage by year.

The median operative duration was 167 minutes (IQR: 131–210). General anaesthesia was used in 60% (95% CI: 52–67.5%) of patients and a combination of general and neuraxial anaesthesia in 38.7% (95% CI: 31.3–46.7%). The use of general anaesthetic only increased from 53.3% in 2005 to 83.3% in 2009, with general and neuraxial anaesthesia, decreasing from 43.3% in 2005 to 16.7% in 2009 as shown in Figure 1. 

The median number of days to ambulation decreased from three days (IQR: 2-3) in 2005 to 1.5 days (IQR: 1-2) in 2009.

### 3.3. VTE Prophylaxis

Pharmacological prophylaxis was used in 98.7% (95% CI: 95.3–99.6%) and mechanical prophylaxis in 99.3% (95% CI: 96.3–99.9%) of patients. LMWH was used in 35.3% (95% CI: 28.1–43.3%) of patients and LDUH in 52.7% (95% CI: 44.7–60.5%). LDUH followed by LMWH was used in 10.7% of patients (95% CI: 6.7–16.6%). The pharmacological prophylaxis dose regimes utilised are shown in Table 1. 

**Figure 2:**
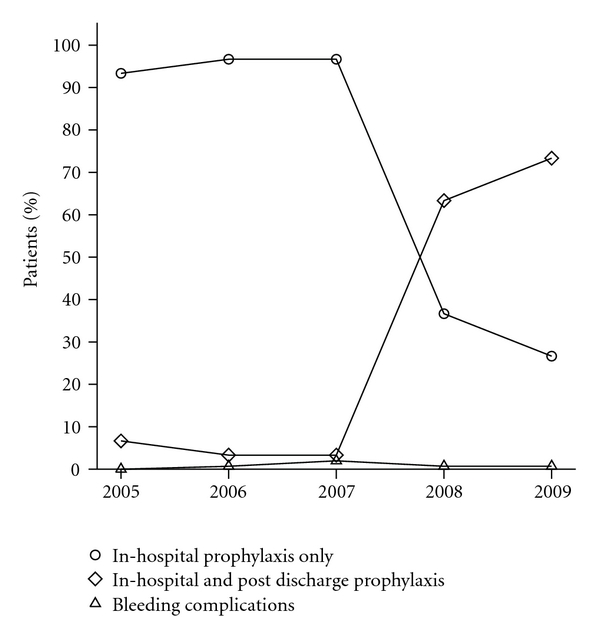
Duration of pharmacological VTE prophylaxis.

The duration of in-hospital pharmacological VTE prophylaxis decreased from 10 days (IQR: 4–12.5) in 2005 to 5.5 days (IQR: 3–8) in 2009 corresponding to decreasing length of stay. Pharmacological prophylaxis was given to 86% (95% CI: 79.5–90.7%) of patients postoperatively only compared to 12.7% (95% CI: 8.3–18.9%) receiving heparin preoperatively and postoperatively. Median time to operation for preoperative LMWH administration was 13 hrs (IQR: 8.0–17.8 hrs) and from operation for postoperatively only administration was 8.5 hrs (IQR: 5.7–23.3 hrs). Median time to operation from preoperative LDUH administration was 17.1 hrs (IQR: 12.2–24.6 hrs) and from operation for postoperatively only administration was 7 hrs (IQR: 5.1–9.3 hrs). The changing pattern over five years (2005 to 2009) in duration of pharmacological VTE prophylaxis in hospital and after discharge, is shown in Figure 2.

ACCP and NHMRC guidelines for VTE prophylaxis recommend colorectal cancer surgery patients receive LMWH or LDUH for at least seven to 10 days postoperatively with consideration of extended prophylaxis for up to 28 days after discharge [7, 8]. During hospital stay, 76% (95% CI: 68.6–82.1%) of patients received the recommended in-hospital VTE prophylaxis for at least seven to 10 days. For patients with a length of stay less than seven days, 52.4% (95% CI: 32.4–71.7%) received extended prophylaxis up to 14 days after discharge. Overall, in the period after discharge, 68.4% (95% CI: 61.6–76.2%) of patients did not receive any VTE prophylaxis, 25.7% (95% CI: 19.6–33.6%) received up to 14 days of VTE prophylaxis and 4.6% (95% CI: 2.3–9.3%) of patients received VTE prophylaxis beyond 14 days and up to 28 days after discharge. The use of VTE prophylaxis after discharge has improved dramatically from 6.7% (95% CI: 1.9–21.3%) in 2005 to 73.3% (95% CI: 55.6–85.8%) in 2009. In terms of VTE prophylaxis regimes on discharge, 25.3% (95% CI: 19.1–32.9%) of patients used LMWH 40 mg daily and equal minorities of 1.3% (95% CI: 0.3–4.7%) patients used LMWH 20 mg daily, LDUH 5000 IU twice daily, or warfarin.

### 3.4. Major Bleeding Complications

Major bleeding complications occurred in 4% (95% CI: 1.9–8.5%) of patients, 3.3% receiving LDUH and 0.7% LMWH (70 patients who were anaemic preoperatively and two patients with unknown preoperative haemoglobin were excluded from analysis).

### 3.5. VTE Incidence

**Figure 3:**
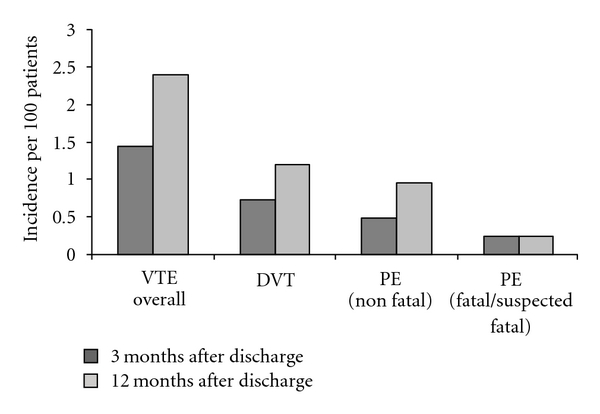
VTE incidence.

From the time of original admission to 12 months after discharge, the overall incidence of VTE was 2.4% (95% CI: 1.3–4.4%). Of these patients, 75% (95% CI: 40.9–92.9%) had a TNM stage greater than three and 62.5% (95% CI: 13.7–69.4%) did not receive VTE prophylaxis after discharge. The median number of co-morbities in this group was 4.5 (95% CI: 2–5.5). The proportion of VTE events at three months and at 12 months is shown in Figure 3 (no VTE events occurred in hospital). DVT occurred in 1.2% (95% CI: 0.5–2.8%) of patients overall, non fatal PE in 0.9% (95% CI: 0.4–2.4%), and suspected or confirmed fatal PE in 0.2% (95% CI: 0.04–1.4%). Both DVT and PE occurred in 0.5% (95% CI: 0.1–1.7%) of patients. The median length of time after discharge to each type of VTE event was 70 days (IQR: 67.8–202.8) for DVT, 57.5 days (IQR: 41–114.3) for non fatal PE, and 8 days (IQR: 8–8) for suspected or confirmed fatal PE. The 30-day mortality rate was 1.9% (95% CI: 1.0–3.7%) with the causes of death listed in Table 2. 

### 3.6. Readmission

Of the 417 patients, 19.4% (95% CI: 15.9–23.5%) experienced at least one hospital readmission in the three months after discharge. At 12 months after discharge, 27.6% (95% CI: 23.5–32.1%) had at least one readmission. VTE accounted for 7.4% (95% CI: 3.4–15.2%), and 8.7% (95% CI: 4.8–15.3%) of readmissions in the first three and 12 months, respectively.

## 4. Discussion

The overall incidence of VTE in patients undergoing colorectal surgery for suspected or confirmed malignancy was found to be 2.4% out to 12 months, with all identified VTE events occurring following initial hospital discharge. More than half of these VTE events (1.4%) occurred within three months of discharge. The incidence of VTE in this study is lower than published figures and may underestimate the true incidence of VTE [10]. Some patients may have been managed at another hospital or by their medical practitioner. Colorectal cancer surgery patients have been shown to be at significant risk of VTE during the period after discharge and appropriate prophylaxis is able to reduce this risk [11–13]. A meta-analysis based on three randomised controlled trials of extended VTE prophylaxis totalling 1,104 major abdominal surgery patients demonstrated a significant reduction in VTE incidence at cessation of extended prophylaxis, where 5.9% of extended prophylaxis patients experienced VTE compared to 13.6% of in-hospital only prophylaxis patients [10]. VTE incidence was examined in two trials and at three months was found to be 6.2% in the extended prophylaxis group compared to 14.3% in the in-hospital only prophylaxis group [10]. This study also demonstrated no significant difference in the incidence of major or minor bleeding with extended prophylaxis, with 3.9% in the extended prophylaxis group and 3.5% in the in-hospital only prophylaxis group [10].

The incidence of major bleeding complications found in this study (4%) is comparable to other studies and despite the difference in incidence of major bleeding complications between LDUH and LMWH in this study (3.3% and 0.7%, respectively) many studies have demonstrated no significant difference in risk of bleeding between LDUH and LMWH [10, 14].

Our patients' median length of stay decreased from 13 days (IQR: 10.3–19.8) in 2005 to 7 days (IQR: 6–11) in 2009 with patients undergoing a laparoscopic procedure having a significantly shorter LOS (*P* < .0001). The time to ambulation after surgery decreased by half, from a median of three days in 2005 to one and a half days in 2009, with earlier mobilisation likely decreasing risk of VTE. This may be partially attributable to a decrease in the use of combination general and neuraxial anaesthesia and an increased use of general anaesthesia alone allowing for earlier ambulation without adjunctive neuraxial anaesthesia. In patients found to have VTE, multiple recognised risk factors were present including advanced-stage cancer, multiple co-morbidities, and operative duration greater than two hours [4]. Chemotherapy is a significant risk factor for the development of VTE in cancer patients and may have contributed to the incidence of VTE after discharge in this study; however, examination of its effect was beyond the scope of this study [5].

In the period 2005 to 2009, the median duration of in-hospital pharmacological VTE prophylaxis fell from 10 days in 2005 to 5.5 days in 2009, corresponding with the fall in median LOS from 13 days in 2005 to seven days in 2009. In contrast, the percentage of patients receiving pharmacological VTE prophylaxis in the period after discharge increased dramatically from 6.7% in 2005 to 73.3% in 2009, with the main increase occurring in the period 2007-2008 which corresponds to the introduction of interventional strategies such as paper reminders placed in clinical notes for ordering of post discharge pharmacological prophylaxis in this high-risk group of patients [15].

A systematic review of strategies to improve VTE prophylaxis in hospitals demonstrated that active interventions such as electronic decision-support systems and paper-based reminders were more effective than passive dissemination of guidelines, although the studies examined were not adequately powered to demonstrate a statistically significant reduction in the incidence of VTE [16].

In conclusion, this study of colorectal surgery patients with suspected or confirmed malignancy demonstrated no in-hospital symptomatic VTE events. While all patients received VTE prophylaxis during admission, the occurrence of the majority of VTE events in the three-month period after discharge and the trend towards decreasing length of hospital stay highlights the importance of VTE prophylaxis in the period immediately after discharge. To minimise the occurrence of VTE after discharge, it is imperative to educate and support clinicians, allied health staff, and patients on the importance of VTE preventative strategies.

## Figures and Tables

**Table 1:** Pharmacological VTE prophylaxis dose regimes.

Pharmacological prophylaxis dose regime	% of patients	% of patients post discharge prophylaxis	12 month VTE incidence (%)	Major bleeding complications (%)
LMWH 20 mg daily	5.3	0.7	0	0
LMWH 40 mg daily	30	16	1.2	0.7
LDUH 5000 IU twice daily	30.7	4.7	1	1.3
LDUH 5000 IU three times daily	3.3	0.7	0	0
LDUH followed by LMWH	10.7	4.7	0.2	0.7
None or inadequate LDUH	20	4	0	0.7

**Table 2:** 30-day mortality cause.

30-day mortality cause	*N*	% of deaths
Acute renal failure	3/8	37.5
Sepsis	3/8	37.5
Cardiac complications	2/8	25
Gastrointestinal haemorrhage	2/8	25
Pulmonary embolism	1/8	12.5
Respiratory failure	1/8	12.5

*A given patient may have more than one cause of death.
